# Identifying and Reducing Systematic Errors in Chromosome Conformation Capture Data

**DOI:** 10.1371/journal.pone.0146007

**Published:** 2015-12-30

**Authors:** Seungsoo Hahn, Dongsup Kim

**Affiliations:** 1 Department of Chemistry, Chung-Ang University, Seoul, South Korea; 2 Department of Bio and Brain Engineering, KAIST, Daejeon, South Korea; Tulane University Health Sciences Center, UNITED STATES

## Abstract

Chromosome conformation capture (3C)-based techniques have recently been used to uncover the mystic genomic architecture in the nucleus. These techniques yield indirect data on the distances between genomic loci in the form of contact frequencies that must be normalized to remove various errors. This normalization process determines the quality of data analysis. In this study, we describe two systematic errors that result from the heterogeneous local density of restriction sites and different local chromatin states, methods to identify and remove those artifacts, and three previously described sources of systematic errors in 3C-based data: fragment length, mappability, and local DNA composition. To explain the effect of systematic errors on the results, we used three different published data sets to show the dependence of the results on restriction enzymes and experimental methods. Comparison of the results from different restriction enzymes shows a higher correlation after removing systematic errors. In contrast, using different methods with the same restriction enzymes shows a lower correlation after removing systematic errors. Notably, the improved correlation of the latter case caused by systematic errors indicates that a higher correlation between results does not ensure the validity of the normalization methods. Finally, we suggest a method to analyze random error and provide guidance for the maximum reproducibility of contact frequency maps.

## Introduction

Understanding the spatial organization of the human genome provides a deeper knowledge on biological processes [1–3]. Chromosome conformation capture (3C) and its related (3C-based) technologies have been used to explore the relationship between spatial genome organization and functions [4–6]. The 3C-based technologies measure the distance between genomic loci as a form of contact frequency [4, 5, 7, 8]. Because of the indirect measure of the distance, identifying and removing systematic errors entangled in the experimental data are fundamental steps to improving the quality of the results [4–6, 8–11].

Various methods have been suggested to remove the biases at the fragment and segment levels [11–14]. Yaffe and Tanay suggested the probabilistic modeling method, where various error sources—such as fragment length, mappability, and GC content—were identified based on the fragment level and corrected based on the probabilistic model [11]. Although this approach is advantageous in that the physical origins of experimental biases are clearly shown, an unknown portion of error sources can become magnified in the processed results. Alternatively, Imakaev et al. suggested an iterative segment-level correction method by equalizing the coverage of each segment from the frequency of double-sided and single-sided reads [12]. This approach avoids systematic errors of complex physical origins but may increase the artificial error caused by physically isolated fragments that have no nearby restriction sites.

In this study, we first obtained an upper limit of correlation (ULC) between experimentally-obtained contact frequency maps by analyzing random error and provided a theoretical method to estimate the ULC. We next described a method by integrating the advantages of both fragment- and segment-level approaches to correct systematic errors. Here, we identified two fragment-level error sources, the local density of surrounding restriction sites (DR) and the local chromatin state (CS). We also suggest methods to remove those and three other previously described systematic errors: the fragment length between closest restriction sites (FL), the mappability score near restriction sites (MS), and the local DNA composition near restriction sites (DC). Among the five systematic errors, FL, DC, and DR originate from the use of restriction enzymes, MS from reference genome mapping, and CS from the heterogeneous nuclear local protein density. Note that the five systematic errors are analyzed at the level of fragment ends, meaning that correction of the errors makes each fragment end have an equal probability of findings. Thus, the following genomic-segment-based analysis should be divided by the number of belonging fragment ends. Finally, we detail the effect of the systematic errors on the results and compare the correction results with those of the previously used method.

## Materials and Methods

### Chromosome conformation capture data

Experimental data from chromosome conformation capture (3C)-based methods were downloaded from the NCBI Sequence Read Archive [(GSE18199 for genome-wide Hi-C and SRA025848 for tethered chromatin capture (TCC)] [4, 10]. Lieberman-Aiden et al. reported on genome-wide long-range interactions between all genomic positions using 3C-based methods on the human lymphoblastoid cell line (GM06990), where sequence paired-end reads were prepared using HindIII or NcoI restriction enzyme digests [10]. Kalhor et al. described data for the human lymphoblastoid cell line (GM12878) by applying an improved technique and suggested a population-based modeling method to build the genome-wide chromosome conformation, where sequence paired-end reads were prepared using the restriction enzyme HindIII [4]. Thus, the Lieberman-Aiden dataset can be used to assess the effect of different restriction enzymes on the results, whereas the Kalhor dataset can be used to examine the effect of different experimental methods on the results when compared to that obtained from the Lieberman-Aiden study. We denote Hi-C HindIII, Hi-C NcoI, and TCC HindIII data as HH, HN, and TH, respectively.

### Mapping sequences

The paired-end reads with the same sequences were removed to reduce PCR duplication error. Sequences containing potential ligation junction sites, such as the sequences containing AAGCTAGCTT for HindIII and CCATGCATGG for NcoI, were trimmed after the junction site (for example, between AAGCT and AGCTT in AAGCTAGCTT for HindIII). We only considered 40 bp from the starting position to simplify the mappability analysis and used only the sequences that contained ≥ 40 bp with less than two unidentified nucleotides. Sequences from both sides of each paired-end read were separately mapped to the reference genome (hg19) using Bowtie2 with the option to exclude multiple aligned sequences with a mapping quality < 30 [15].

### Screening paired-end reads

We analyzed the mapping sequences based on restriction sites because normal mapping positions of both sequences from a paired-end read are located near restriction sites. For the analysis, each restriction site was orderly indexed from the beginning of the reference genome, where the numbers of restriction sites in the human reference genome (hg19, excluding the Y chromosome) are 830,194 and 744,516 for HindIII and NcoI, respectively. Each restriction site contains two fragment ends according to the direction on the reference genome, and the two fragment ends were treated separately. Because the analysis of sequences for systematic error corrections has been performed near fragment ends, we defined the location of sequence blocks according to each fragment end for convenience. If a sequence block is located before a fragment end or after a fragment end, the block was defined as a BFE or AFE sequence, respectively.

Each mapping position of the sequences was then represented as a label constituting three elements: an index of the closest restriction site located in the sequencing direction, a genomic distance from the restriction site to the starting position of the mapping sequence, and a sign designating the fragment end belonging to the restriction site with a "+" sign for the reference genome direction and a "-" sign for the opposite direction. Thus, a paired-end read can be expressed as {(*r*_1_,*d*_1_,*s*_1_),(*r*_2_,*d*_2_,*s*_2_)}, where *r*, *d*, and *s* denote an index of the closest restriction site, the genomic distance, and the direction sign, respectively. In the label for each mapping position, a fragment end is uniquely described by two elements, *r* and *s*, without *d*. Because only the paired-end reads with a sequence length ≤ 500 bp (or *d*_1_ + *d*_2_ ≤ 500 bp) are valid in our analysis, one-side sequences from paired-end reads must have a genomic distance (*d*) ranging from 40 bp to 460 bp [11, 12].

The coverage value of each fragment end in the experimental data was evaluated by counting the total number of paired-end reads containing the fragment end. We excluded some fragment ends based on their coverage value, where we fit the distribution of all coverage values using a standard Gaussian model and then simply excluded the extreme cases (standard score > 3). Paired-end reads containing the excluded fragment ends were removed.

**Fig 1:**
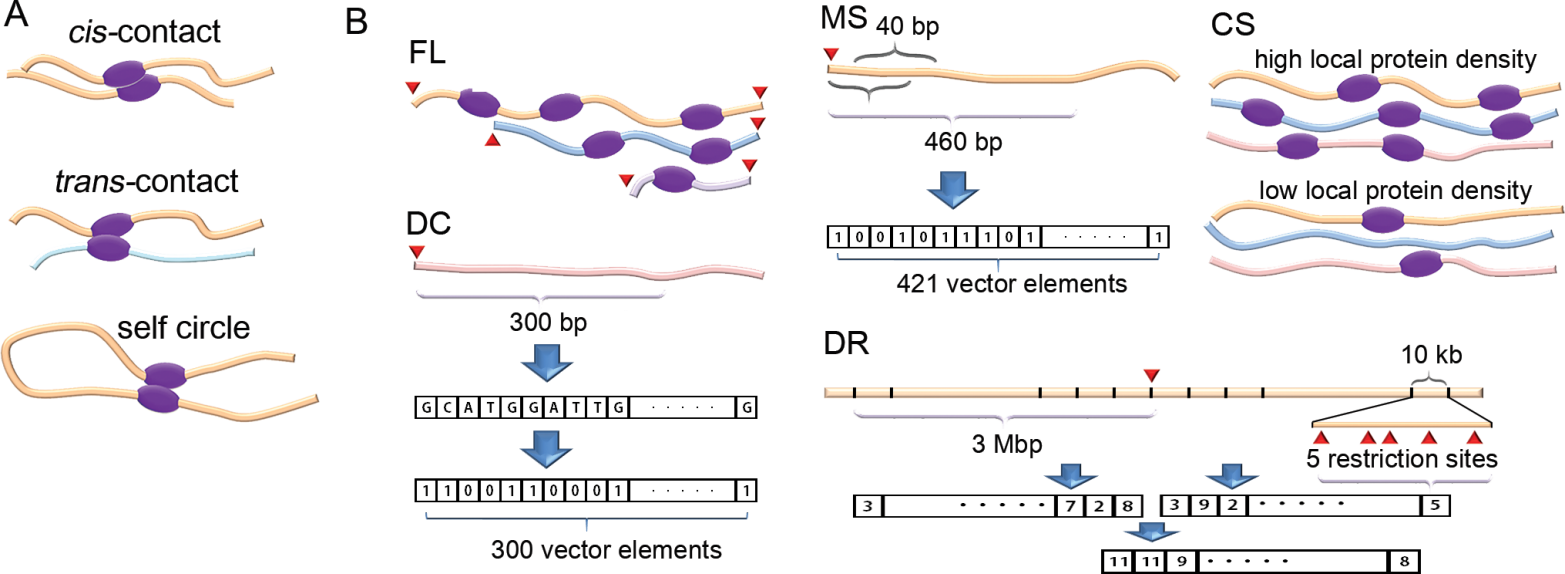
Conceptual diagrams of contacts and systematic errors. (A) Two different types of contacts, *cis*-contact and *trans*-contact, are shown. A self-circle denotes a *cis*-contact between the closest restriction sites. (B) Conceptual diagrams of five systematic errors are shown, where fragment ends are denoted as red triangles, thick curved lines with different colors denote DNA chains from different chromosomes, and purple oval figures denote DNA-binding proteins. FL: a DNA chain with a longer fragment length has a greater chance of being bound with nearby chains. MS: all possible 40-bp sequences for each fragment end are mapped into the reference genome, and the mapping results are indexed. DC: fragment ends with different DNA compositions show different characteristics under an experimental condition. DR: denser nearby restriction sites yield a higher chance of meeting the target fragment end. The number of restriction sites belonging to each genomic segment is indexed into a vector. CS: the local density of the proteins that bind nearby DNA chains has an effect on the coverage value.

The resulting paired-end reads were divided into five groups by following the method used in reference [12]. The five groups are *cis*-contact, *trans*-contact, self circle, single end, and dangling end (Fig 1A). The coverage value of each fragment end was recalculated by counting the valid one-side sequences from all five groups, and these coverage values were used for the correction of systematic errors.

### Explanatory variables for systematic errors

We corrected systematic errors based on the coverage values of fragment ends (the systematic errors being FL, MS, DC, DR, and CS). Conceptual diagrams of the systematic errors are shown in Fig 1B. We developed the following explanatory variables for the correction of the systematic errors.

For FL, digesting the two closest restriction sites in a chromosome generates a restriction fragment with two fragment ends. The genomic length of the fragment is used as an explanatory variable of the two fragment ends.

For MS, we extracted a 460-bp BFE sequence from each fragment end in the genome and obtained 421 small 40-bp sequences from the 460-bp sequence by shifting a 40-bp window from the fragment end. The small sequences were then individually mapped onto the reference genome. Thus, the mapping sequences for a fragment end can be represented as a vector with 421 elements, where an element value is 1 for a uniquely mapped sequence and 0 for other cases. For the evaluation of the previous mappability score, we added all the elements in each vector, divided the sum by 421, and used the result as an explanatory variable [11]. We improved the score by implying two findings, that the population of the genomic distances (the *d* values in the mapping position label) in the experimental data is unevenly distributed according to their position from the fragment end (S1B Fig) and that the number of 40-bp sequences extracted from the genome sequence depends also on their position because of the existence of short restriction fragments of < 460 bp. A probability vector was built by dividing the population by the number of 40-bp sequences according to their position and normalizing the sum of elements to be 1. A new mappability score is a vector dot value of the mappability vector with the probability vector. The score was used as a new explanatory variable for MS.

For DC, we extracted a 300-bp BFE sequence from each fragment end in the genome and then represented the sequence as a vector with 300 elements, where an element value is 1 for G or C and 0 for A or T. These elements were used as explanatory variables for the multiple linear regression.

For DR, we analyzed the BFE and AFE sequences at each restriction site < 3 Mbp for each direction, divided each sequence into 300 sectors with a 10-kb genomic length, and counted the number of restriction sites that belong to each sector. This method yielded two vectors with 300 elements for each restriction site. We assumed that the coverage value of a fragment end is mainly affected by the genomic length between the fragment end and nearby restriction sites. We combined the two vectors into one using the vector sum. The 300 elements in a vector were used as explanatory variables.

For CS, chromatin states classified by Ernst et al. were used, where 15 classes of chromatin states were assigned to the human genome [16]. For each fragment end, we analyzed the chromatin state composition of a 1000-bp BFE sequence by counting the number of nucleotides belonging to each class of the chromatin state, making a vector with 15 elements containing the number of nucleotides as its elements and normalizing the vector to have a magnitude of 1.

Variance inflation factors (VIF) of explanatory variables were evaluated to check the multicollinearity using R packages, where the factor is close to 1 for independent variables and a large value for the variables dependent on other explanatory variables [17–19]. The 15 explanatory variables for CS show high multicollinearity (VIF of 200). To reduce the multicollinearity, we removed 1 variable classified as the "heterochromatin state," where a regression coefficient of the variable was the smallest and the removal of the variable had a slight effect on other regression coefficients. To evaluate the standard deviation of the coefficients, we systematically increased the length of BFE sequences from 500 bp to 5000 bp by 500-bp intervals. VIFs of the 14 explanatory variables were less than 3 and used as explanatory variables for CS. VIFs of all 616 explanatory variables were evaluated together (S2 Fig). In our case, all VIFs are still less than 3, indicating that the explanatory variables are independent.

### Multiplicative systematic errors

The free energy change for a coverage value (*C*) of a fragment end can be described as Δ*G*_*C*_ = −*RT* ln(*α*_*C*_ *C*), and the free energy changes for the five systematic errors are Δ*G*_*X*_ = −*RT* ln(*α*_*X*_ *X*), where *X* is a quantity caused by one of the five systematic errors, and the quantity is linearly proportional to the coverage value. We considered the quantity an abundance caused by each systematic error. For convenience, we denote the quantities caused by the five systematic errors, FL, MS, DC, DR, and CS, as *L*, *M*, *D*, *R*, and *S*, respectively. Summation of the free energies results in multiplicative systematic errors as *C* = *α*⋅*L*⋅*M*⋅*D*⋅*R*⋅*S*, where α is a scaling constant to adjust the arbitrary size of the experimental data and is not changed according to a fragment end.

### Coefficients of explanatory variables

First, we investigated the effect of FL on the coverage value of each fragment end. To reduce the bias caused by other systematic errors, we only used a group of fragment ends with an MS of 1. Because of the scarcity of longer FLs, the common logarithm of FLs was used to classify fragment ends into several bins ranging from 0 to 4 by 0.02 intervals on the logarithm scale. The average of the coverage values of each bin was evaluated and normalized by the maximum value among the averages, which were subsequently used as the abundance caused by FL. We found that the averages were almost constant in the range from 2kb to 10kb. This length range was used to evaluate the regression coefficients of other explanatory variables as below.

Second, coverage values were collected according to MS and their average value was evaluated. The average values are linearly correlated with MS, meaning that MS directly represents the abundance. We used an MS of 1 in the analysis of DR and DC to reduce MS-caused error.

Third, the effect of variance in DC to the abundance is considered in a physical concept that each nucleotide in the DNA sequence contributes energetically to the abundance. Thus, the natural logarithm of the abundance is directly proportional to the linear combination of the DNA composition from a fragment end. The regression coefficients for DC were evaluated using the multiple linear regression with the natural logarithm of the abundances as dependent variables.

Fourth, the abundance caused by DR was evaluated based on the 300 explanatory variables. Each variable for DR denotes the number of restriction sites. Thus, each variable can independently contribute to the abundance with a weighting factor to consider the effect of the different locations of each element to the contact probability with the target fragment end. The weighting factors were evaluated using the multiple linear regression. Because of the multicollinearity between DR and DC, the simple product of *R* and *D* for the evaluation of the coverage value causes an error. We solved the multicollinearity using the iterative method explained in the next section.

The above four systematic errors reflect artificial effects caused by a restriction enzyme and by a mapping error to the reference genome. If we can successfully remove the artificial effects, then the only remaining error source that affects the coverage value of each fragment end is the heterogeneous density of proteins, which entangle nearby DNA chains after the addition of formaldehyde. We conjectured that the density of proteins depends on the local chromatin states. The 14 variables for CS were used as independent variables, and the abundance after removing the four systematic errors was used as a dependent variable for the multiple linear regression. In the regression, we used the fragment ends with MS > 0.5 and FL between 460 bp and 10 kb.

### Iterative method for entangled systematic errors

Errors attributed to DC and DR are not separable. We developed an iterative method to evaluate the parameters for each systematic error using the multiple linear regression. For this, we assumed that each vector element for DR was additively proportional to the abundance and that each vector element for DC was additively proportional to the logarithm of the abundance, as explained in the previous section. Let us assume that *C*_*i*_, *R*_*ij*_, and *D*_*ij*_ denote the abundance, *j*th element of the DR vector, and *j*th element for the DC vector of the *i*th fragment end, respectively. The purpose of the iterative method is to evaluate the contribution of DR and DC to the coverage value. Additionally, let us denote *R*_*i*_ and *D*_*i*_ as the contribution of DR and DC to the coverage value of the *i*th fragment end, meaning that *C*_*i*_ = *α*⋅*R*_*i*_⋅*D*_*i*_. The regression coefficients for independent variables for the multiple linear regressions are evaluated as follows:

1. Perform the multiple linear regression for the equation
  $$\ln\left(C_{i}\right)=d_{0}+d_{1}D_{i1}+d_{2}D_{i2}+\cdots +d_{n}D_{in}.$$
  The regression yields a set of regression coefficients *d*_*j*_ and the estimated dependent variable 〈ln(*C*_*i*_)〉. If we denote the predicted value of *D*_*i*_ as ${\hat{D}}_{i}$, then ${\hat{D}}_{i}$ has a relation with the estimated dependent variable as $\ln\left({\hat{D}}_{i}\right)=\left\langle \ln\left(C_{i}\right)\right\rangle$.
2. Because we want to evaluate the coverage value as a form of a product of *R*_*i*_ with *D*_*i*_, division of the coverage value by ${\hat{D}}_{i}$ is proportional to *R*_*i*_. Performing the multiple linear regression for the equation
  $$C_{i}/{\hat{D}}_{i}=r_{0}+r_{1}R_{i1}+r_{2}R_{i2}+\cdots +r_{n}R_{in}.$$
  yields a set of regression coefficients *r*_*j*_ and the estimated dependent variable $\left\langle C_{i}/{\hat{D}}_{i}\right\rangle$. If we denote the predicted value of *R*_*i*_ as ${\hat{R}}_{i}$, then ${\hat{R}}_{i}$ is equal to $\left\langle C_{i}/{\hat{D}}_{i}\right\rangle$.
3. From the set of ${\hat{R}}_{i}$ values, the ${\hat{D}}_{i}$ values can be refined by performing the multiple linear regression for the equation $\ln\left(C_{i}/{\hat{R}}_{i}\right)=d_{0}+d_{1}D_{i1}+d_{2}D_{i2}+\cdots +d_{n}D_{in}$. If we denote the estimated dependent variable as $\left\langle \ln\left(C_{i}/{\hat{R}}_{i}\right)\right\rangle$, then ${\hat{D}}_{i}$ can be evaluated from the relation $\ln\left({\hat{D}}_{i}\right)=\left\langle \ln\left(C_{i}/{\hat{R}}_{i}\right)\right\rangle$.
4. Iterate the steps from 2 to 3 until the standard deviation between the actual and estimated coverage values converges.

If a percent change of the standard deviation after an iteration was less than 0.0001, we stopped the algorithm. The dependence of the product ${\hat{R}}_{i}\cdot {\hat{D}}_{i}$ on the initial values used in step 1 is not significant (data not shown).

### Normalized contact frequency maps

A correction factor of *n*th fragment end *C*_*n*_ is equal to the product of all systematic errors, or *C*_*n*_ = *α*⋅*L*_*n*_⋅*M*_*n*_⋅*R*_*n*_⋅*D*_*n*_⋅*S*_*n*_. We evaluated a normalization factor of the *i*th genomic segment by summing all correction factors of the fragment ends consisting of the genomic segment as $N_{i}=\sum_{n\in i}C_{n}$. If we define *C*_*ij*_ as the raw contact frequency between the *i*th and *j*th genomic segments, then normalized contact frequency is evaluated as

$$NC_{ij}=\frac{C_{ij}\cdot {\bar{N}}^{2}}{N_{i}\cdot N_{j}}$$

where *NC*_*ij*_ and $\bar{N}$ denote a normalized contact frequency and the mean of *N*_*i*_s, respectively. To reduce an artificial effect of the magnification from small normalization factors, we considered only the genomic segments with $N_{i}/\bar{N}$ > 0.4 for the comparison between the normalized contact frequency maps.

## Results

The experimental error in 3C-based data consists of random and systematic errors. Random error originates inherently from the limited size of paired-end reads because the limited size results in their random inclusion. This error cannot be fixed by processing the data, and it affects the resolution and quality of contact frequency maps. In contrast, systematic errors can be corrected by analyzing their physical origins. Systematic errors include errors due to FL, MS, DC, DR, and CS.

### Random error

High reproducibility is critical for experimental results. To quantify the reproducibility, we used Pearson's correlation coefficient between contact frequency maps. Depending on the contact frequency map, there is a ULC with other maps caused by the data size. Here, we describe a method to estimate the ULC. Contact frequency maps can be notably different from each other due to minute differences in experimental conditions. Although one may generate the experimental results by identical means, there is still stochastic error in experimental data acquisition. An inevitable stochastic error source is a limited sequencing depth that causes an uncertain break in data acquisition. In this demonstration, we only address the stochastic error of technical replicates from the same experimental result. Because the error from minute differences in experimental conditions is independent of the stochastic error, inclusion of both errors will generate a lower correlation between experimental results than when including only stochastic error. Thus, analysis of the experimental results containing only the stochastic error indicates the ULC of a contact frequency map when one obtains the map from another experiment. In this study, we report on Pearson's correlation coefficients between contact frequency maps and compare the results to their ULCs.

#### Evaluation of the ULC

Spatial distances between genomic segments are indirectly measured as a relative frequency of paired-end reads that connect the segments. A contact frequency map can be obtained as a result, consisting of many square blocks on a two-dimensional surface. Each block displays the individual contact information between two genomic segments. Paired-end reads from 3C-based methods then appear as points on the block. The number of points on each block, or a contact frequency, follows an absolute probability that represents the spatial proximity of two genomic segments. To measure the reproducibility of a contact frequency map, we randomly divided experimental data into two groups, which were then used to evaluate two contact frequency maps. Pearson's correlation coefficient of off-diagonal elements between the two contact frequency maps was used as an experimental value for the ULC. The ULC of the contact frequency maps was obtained from one of the two maps using the mean and variance of contact frequencies as represented in Equation 2 in S1 Text.

**Fig 2:**
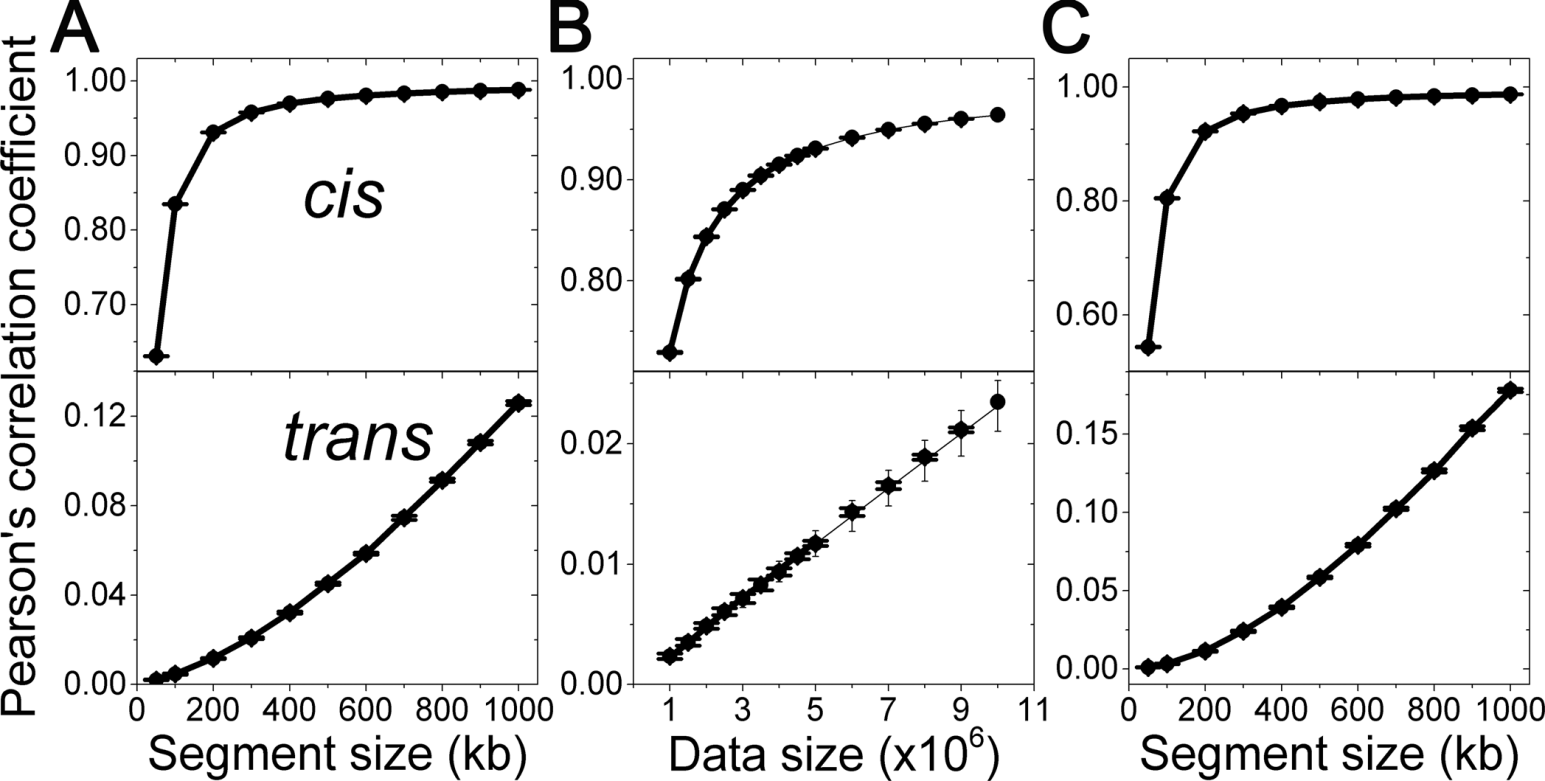
Random error effect. Pearson's correlation coefficients of off-diagonal elements between contact frequency maps from TH data are plotted. In the off-diagonal elements, *cis*- and *trans*-contacts are separately considered. Mean values of experimentally-determined ULCs are plotted as thick lines. Theoretically-determined ULCs are plotted as solid circles with error bars. (A) The segment size effect on ULC was evaluated at the data size of 5,000,000 points. (B) The data size effect on ULC was evaluated based on the fixed segment size of 200 kb. ULCs for larger data sizes were predicted from the data size of 1,000,000 and plotted as thin lines with error bars. (C) ULCs for normalized contact frequency maps were evaluated at the data size of 5,000,000 points. Here error bars denote the standard deviation obtained from the analysis of 10 data sets.

For any given experimental setup, the segment size and data size would be the determinant factors in a map’s reproducibility. First, we systematically changed the segment sizes from 50 kb to 1 Mbp at the fixed data size of 5,000,000 points for TH data. In Fig 2A, ULCs are plotted as solid circles with error bars, while the corresponding experimental values are plotted as thick lines. ULCs accurately predict the experimental values for both *cis*- and *trans*-contacts. Second, we systematically increased the data size from 1,000,000 points to 5,000,000 points by 500,000-point intervals at a segment size of 200 kb. In Fig 2B, similar to the previous case, ULCs matched the experimental data precisely.

#### Estimation of the ULC for a larger data size

The ability to predict the ULC for a larger data size from a smaller constituent would be advantageous with respect to experimental methodology. Pearson's correlation coefficients for larger data sizes were measured experimentally, and corresponding ULCs were predicted using the mean and variance of the data size of 1,000,000 (see Equation 3 in S1 Text). In Fig 2B, the experimentally-measured ULCs, theoretically-evaluated ULCs, and predicted ULCs from a small data size are plotted as thick lines, solid circles, and thin lines, respectively. The predicted ULCs from a 10-times-smaller data size matched both the experimentally-measured ULCs and the theoretically-evaluated ULCs precisely.

#### ULC for normalized contact frequency maps

A contact frequency between two genomic segments can be influenced by many error sources. Therefore, contact frequency maps have been normalized by various factors for a meaningful comparison in the majority of cases. We detail a method of how to obtain the ULC for a normalized contact frequency map in Equation 4 in S1 Text. For this, we systematically changed the segment size of a contact frequency map, compared the experimentally measured ULCs with the corresponding ULCs and found them to be in agreement (Fig 2C).

### Systematic errors

In contrast to random errors, systematic errors can be corrected by proper normalization methods. We suggest correction methods for five different sources of systematic errors: FL, MS, DR, DC, and CS. Of these, DR and CS are newly identified sources, while the other three have been reported previously [11]. Restriction enzymes cut specific sites throughout the genome by recognizing specific palindromic sequences, generating a great number of fragments in the process. Characteristic properties of each fragment—the heterogeneous distribution of restriction sites, quality of the reference genome, and unique mapping sequences—cause four of the systematic errors (excluding CS, which originates from the properties of the cell nucleus). Conceptual diagrams of the systematic errors are shown in Fig 1B. Each systematic error takes effect on the coverage value of each fragment end.

Let us explain the five different sources of systematic errors. First, each fragment has a different length and different probability to be chemically linked to an adjacent DNA chain, which is described as the systematic error due to FL. Second, each fragment contains a specific sequence and exhibits its own unique mapping ability (mappability) to the reference genome, where MS is linearly correlated to the coverage value. Third, the DC of each fragment end causes some experimental bias because each nucleotide contributes energetically to a binding ability for unknown targets. Fourth, the probability of a fragment end coming into contact with another one is proportional to the density of nearby fragment ends, which is the physical origin for DR. Last, the local protein density in a chromosome has a relation with CS. Higher local protein density causes more connections between DNA chains in that region.

For the evaluation of contributions of the five systematic errors to the coverage value of each fragment end, we assumed that the errors contribute individually to the coverage value of each fragment end as a form of free energy change, Δ*G* = −*RT* ln (*αP*), where *P* is a finding probability, and *α* is a constant with the gas constant *R* and temperature *T*, meaning that a higher coverage value has a larger free energy change. This connection between the free energy change and finding probability has been used in previous studies [20, 21]. In the free energy equation, the finding probability is linearly proportional to the coverage value of a fragment end. The coverage value can be increased in two ways: negative free energy change and increased finding probability. Among the systematic errors, DR and DC have multiple explanatory variables. Each element in DR denotes the number of restriction sites in a given genomic sector, and thus, each element increases the coverage value of the fragment end additively because the coverage value is proportional to the number of surrounding restriction sites. On the contrary, each element in DC denotes a different type of nucleotide and each nucleotide may bind to some unknown materials with a certain interaction energy, where the interaction energy increases the coverage value. This is the suggested physical model for the DC contribution, where each nucleotide contributes to the coverage value as an additive interaction energy. The two quantities affect the coverage value with different mechanisms, but the quantities are integrated using the free energy equation.

#### Systematic error: FL

**Fig 3:**
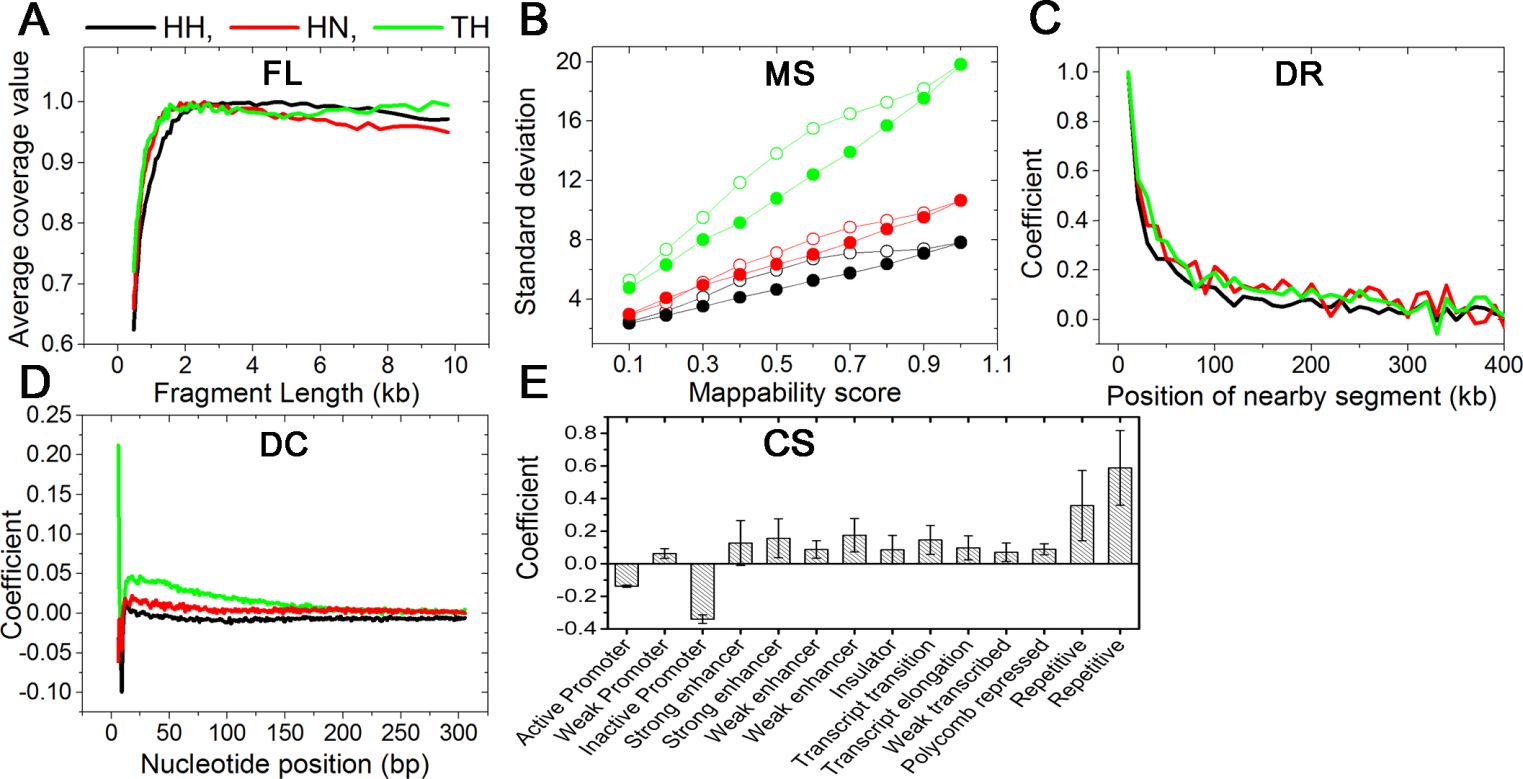
Explanatory variables and coefficients for various systematic errors. (A) A fragment length of a fragment end is an explanatory variable of FL, and an average coverage value is a coefficient of the explanatory variable. (B) The standard deviations of the coverage values classified by old mappability scores and new mappability scores were plotted as open circles and solid circles, respectively. (C) The regression coefficients for DR from the iterative method are plotted according to the position of nearby segments. (D) The regression coefficients for DC are plotted according to the nucleotide positions from fragment ends. (E) The regression coefficients for CS were measured using 14 explanatory variables, where the variables denote 14 different chromatin states classified by a previous study. The positive value of a chromatin state means that the chromatin state contains more proteins for binding nearby DNA chains. The error bars denote standard deviations of the coefficients from three experimental results.

Coverage values are systematically affected by FL. A longer fragment has a greater chance of matching a ligating partner in 3C-based experiments. Thus, it is expected that a longer fragment may have a larger coverage value than a shorter one. In S3 Fig, the three experiments gave different patterns in individual categories of paired reads, such as *cis*-contact, *trans*-contact, self-circle, dangling-end, and single-end, where average contact frequencies of fragment ends consistently go up and down as FL increases. On the contrary, the mean coverage values show an expected pattern for FL. The mean coverage value increases rapidly up to 2 kb and flattens at approximately 10 kb, as shown in Fig 3A. Because of the consistency between experimental results and physical expectation, we used the mean coverage values as the abundance of a fragment end caused by FL.

#### Systematic error: MS

We denoted the mappability of each fragment end as a vector, where the mean of the vector elements was used for MS in the previous method [11]. We grouped fragment ends according to the previous MS and evaluated the mean of their coverage value. The previous MSs are linearly correlated with the mean coverage values (S1 Fig). We developed new MS by considering the difference in experimental conditions. Following the new MS, we evaluated the mean of the coverage values. The new MSs are also linearly correlated with the mean coverage values, similar to the previous MS. However, the variances in the coverage values grouped by the new MS are consistently smaller than those by the previous MS, as shown in Fig 3B. The lower variances demonstrate that the new MS predicts fragment mappability more accurately than the previous one.

#### Systematic error: DC

We represented the DC of each fragment end by a vector. We assume that each nucleotide in the sequence contributes independently to the abundance via its binding energy; in other words, the sum of a nucleotide’s binding energies is proportional to the logarithm of the abundance. Vectors for all fragment ends were fitted to a logarithm of corresponding frequencies using multiple linear regression. The correlation improves rapidly up to a threshold (200 bp) where it then becomes saturated (S4 Fig).

#### Systematic error: DR

We represented the DR of a fragment end by a vector. The vectors were fitted to their corresponding abundance using multiple linear regression. It is readily apparent that including a wider range of nearby sequences improves the correlation results. Fitting results were then plotted according to their covering genomic length from a target restriction site in S4 Fig, where the correlation increases rapidly up to 1 Mbp and becomes nearly saturated at approximately 2 Mbp.

#### Systematic error: CS

A remaining source of coverage value variation is the binding probability of any fragment with adjacent DNA chains. Various proteins in a nucleus tie adjacent nucleotides after formaldehyde fixation. Thus, the coverage value variation of a fragment end after removing four systematic errors—FL, MS, DC, and DR—is proportional to a local density of the proteins. To quantify a local density of the proteins, we used the local chromatin state as independent variables for protein density because the local chromatin state affects the density. We used 15 categories of chromatin state reported from a previous study [16]. In our model, we excluded the "heterochromatin state" variable among the 15 categories to reduce the multicollinearity between independent variables.

Regression coefficients for 14 categories of chromatin states were evaluated. To check the robustness of the method on the selection of a genomic length for CS explanatory variables, we prepared 10 different chromatin state vectors for each fragment end by systematically extending BFE sequences from 500 bp to 5000 bp by 500-bp intervals. Multiple linear regression for each chromatin state vector was performed, and its regression coefficients were evaluated. Standard deviations of the regression coefficients are marked as error bars on the bar graph in S5 Fig. The small standard deviation indicated that the selection of a genomic length for CS explanatory variables took a little effect on the results. For each chromatin state, three experimental results gave the same sign of direction. The mean and standard deviation of 14 regression coefficients are plotted as a bar graph in Fig 3E.

At the level of fragment ends, we compared the abundance caused by CS between three experiments. Because different enzymes gave different restriction sites, comparison at the fragment level is impossible. Thus, we compared the abundance for the three experiments at the segment level. The correlation between experimental methods ranges from 0.521 to 0.878 for 50-kb segments and from 0.598 to 0.930 for 1-Mbp segments.

#### Multicollinearity between DC and DR

We developed the iterative method to reduce the multicollinearity between DC and DR. The method gave regression coefficients for DC and DR simultaneously. In Fig 3C, the coefficients for DR are plotted. The segments located in closer proximity exhibit a larger value than those spaced farther apart, as explained in a previous study [22]. Interestingly, the three experiments all show a similar patterning with Pearson's correlation coefficients > 0.8 for all cases, even when we exclude the contribution of the largest value (inclusion of the value dramatically increases the correlation coefficients).

In Fig 3D, the regression coefficients for DC are plotted according to their relative positions from the fragment ends. Note that positions proximal to the fragment end exert a stronger effect on the results; however, the parameters do not exhibit a clear common pattern and correlation between experiments.

#### Effect of systematic errors on the results

**Fig 4:**
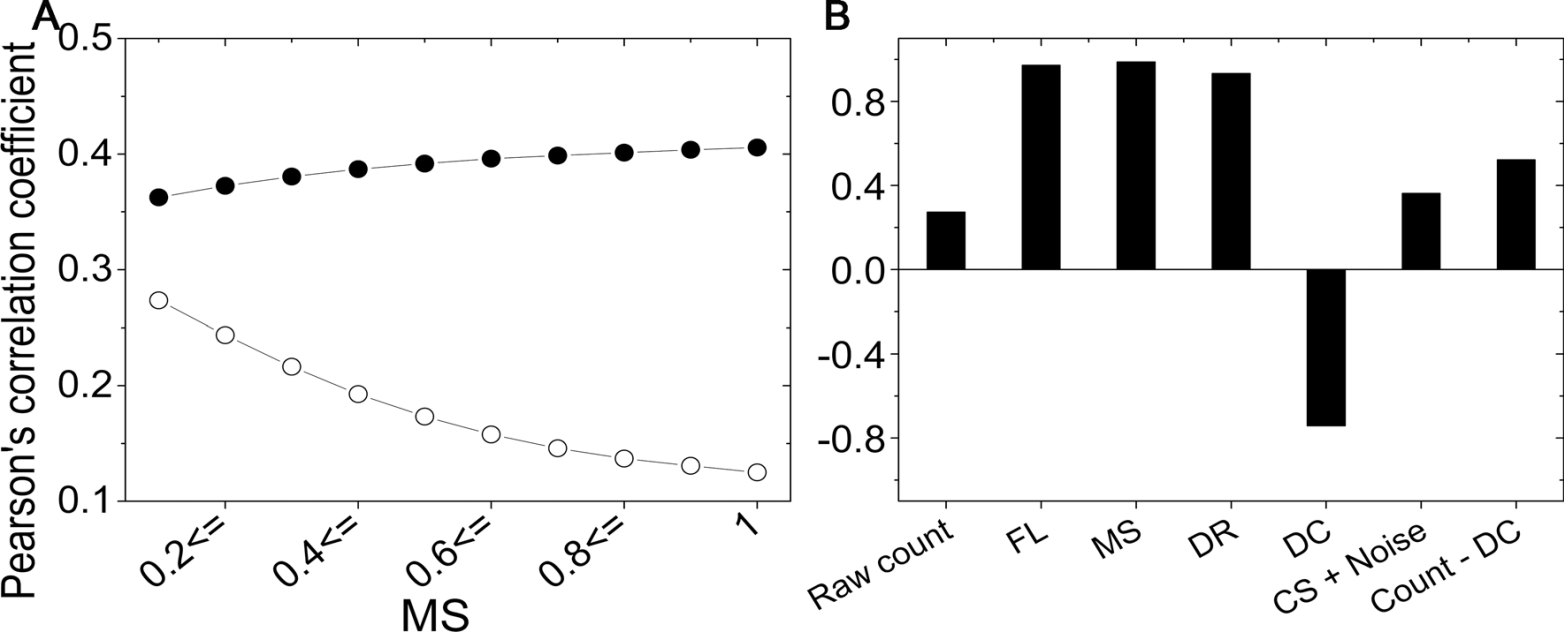
Systematic errors increase the correlation. Comparison results at the fragment level between HH and TH data are shown. (A) The correlation coefficients between raw coverage values are plotted as open circles, and those between corrected values (CS + noise) are plotted as solid circles. (B) The correlation coefficients between correction factors from two experimental results are shown as a bar graph.

Systematic errors may increase the correlation observed between experimental results if they share the same direction of bias. For example, correlations between the experimental results increase when the mappability bias is not corrected. To show the effect of the bias on the results, we evaluated five correction factors of each fragment end for HH and TH data, to compare the data at the fragment level. First, we compared the raw coverage value between experimental data by adjusting a criterion of MS, where we only include fragment ends with an MS larger than the criterion. In Fig 4A, Pearson's correlation coefficients are plotted as circles according to the MS criterion, where an MS ≥ 0.1 has more bias than an MS of 1.0, and the number of participated fragment ends for an MS of 1.0 is 61% of those of an MS ≥ 0.1. From the comparison results, we observed that the correlation between raw coverage values for more biased data (MS ≥ 0.1) is higher than for less biased data (MS of 1.0), as shown in the open circles in Fig 4A. For comparison, we removed four systematic errors in the raw frequency by dividing the frequency by the four correction factors. Thus, the value after the correction involves only CS and noise. Contrary to the raw coverage values, the correlation between corrected values shows an invariance with the addition of biased data, as shown in the solid circles in Fig 4A.

For all fragment ends with MS ≥ 0.1, we compared the abundance between the two experiments at the fragment level. The comparison results are shown in Fig 4B. The systematic errors from FL, MS, and DR show a strong positive correlation; however, DC shows a negative correlation, indicating that the DC is influenced by the experimental setup. After correcting the four systematic errors, the correlation coefficient increases from 0.27 to 0.36. Notably, the addition of systematic errors—except DC—improves the correlation to 0.52.

#### Normalized contact frequency map

The contact frequency maps for three sets of experimental data were prepared based on 1-Mbp segments. The maps were normalized to reduce systematic errors, which improved a correlation between contact frequency maps (S6 Fig). To compare the normalization performance of our method with the previous method, we evaluated the normalized contact frequency maps using three correction factors, FL, MS, and DC, where DC partly contained the effect of DR because of multicollinearity. This normalization method simulates the method suggested by Yaffe and Tanay [11] and is compared to our method, which uses all the correction factors. The difference between the maps from the two methods comes from the different correction factors.

First, we compared the correction factors used for the two methods at the fragment level. The correlation coefficients between the correction factors were 0.96 for HH, 0.89 for HN, and 0.98 for TH. Although, the high similarity between correction factors made little difference between normalized contact frequency maps (S7 Fig), the correction factors for our method gave a higher correlation coefficient with their coverage values compared to the previous method by 4.7% for HH, 14.6% for HN, and 3.4% for TH.

Because of the different normalization factors, ULCs for normalized contact frequency maps from the two methods were different from each other; thus, the correlation between contact frequency maps should be evaluated relative to their ULC, where we used a ratio of the correlation value to its corresponding ULC as a performance of the normalization method.

In Table 1, we compared the performance between methods. For the comparison between the experimental results from different restriction enzymes, normalization of the raw data improved the performance. For *cis*-contacts, performances for HH and HN increased from 76 with raw data to 95 with the previous method and 89 with the new method; those for HN and TH increased from 81 with raw data to 97 with the previous method and 96 with the new method. For the comparison between the experimental results from the same restriction enzyme, normalization of the raw data decreased the performance. For *cis*-contacts, the performance for HH and TH decreased from 97 with raw data to 91 with the previous method and 91 with the new method. For *trans*-contacts, normalization of the raw data improved the performance significantly.

**Table 1:** Pearson's correlation coefficients between contact frequency maps.

		Raw			Previous method^a^			New method		
Data		Exp.	ULC^b^	Ratio (%)	Exp.	ULC	Ratio (%)	Exp.	ULC	Ratio (%)
*cis*	HH /HN	0.746	0.984	76	0.924	0.978	95	0.869	0.977	89
	HH /TH	0.955	0.986	97	0.896	0.984	91	0.899	0.984	91
	HN /TH	0.806	0.990	81	0.951	0.985	97	0.948	0.984	96
*trans*	HH /HN	-0.015	0.258	-6	0.136	0.143	96	0.137	0.148	93
	HH /TH	0.101	0.180	56	0.134	0.158	84	0.149	0.173	86
	HN /TH	0.135	0.235	57	0.096	0.117	82	0.089	0.116	77

^a^ Yaffe's method for normalization of contact frequency maps.

^b^ We used ULC by evaluating a geometric mean of ULCs from both contact frequency maps

## Discussion

Experimental data obtained from 3C-based methods can be affected by various errors from the restriction enzyme, reference genome, and local chromatin state. In this study, we characterized two new systematic errors that result from DR and CS; furthermore, we provided clear physical concepts of the two systematic errors and showed a common pattern amongst the three analyzed experimental results. We could also identify the previously reported systematic errors and succeed in correcting for these using the methods described. Thus, there are five systematic errors, FL, MS, DC, DR, and CS, where the reference genome-derived error is MS, the local chromatin state-derived error is CS, and the restriction enzyme-derived errors are FL, DC, and DR. In addition, we introduced the role of random error to determine the ULC. We provided several equations to address the effect of random error on the results. Using these equations, it is now possible to estimate the required data size to obtain a contact frequency map with a specific segment size and reproducibility.

Contribution of the dangling end and self-circle to normalization of a contact frequency map has been ignored in previous studies [11, 12]. Without these contributions, we could not obtain the desired relationship between FL and the coverage value of paired-end reads. One might expect that longer genomic fragments would have a higher coverage value than its shorter counterpart because a longer one has a higher possibility of being linked to other nearby DNA chains. Thus, the desired pattern is that the coverage value rapidly increases initially and saturates as the length increases. This saturation occurs because the binding position between DNA chains distantly located from fragment ends is similar to two fragment ends with a longer chain length, where the longer chain length diminishes the meeting chance between the two fragment ends [23]. The desired pattern was obtained from the case after summing all contributions.

We suggested new methods to correct systematic errors at the fragment level. First, gathering fragment ends according to the new MS generated better results compared to the previous method in the sense that the variance of the coverage values of the gathered fragment ends is lower. Second, we identified two new systematic errors. It is known that contact frequencies are highly affected by closely located genomic loci [22]. This pattern is also apparent in the effect of DR, where all three experiments commonly show a larger effect of closely located genomic loci on the coverage value, in spite of using different restriction enzymes. After correction of the enzyme-derived error and reference-genome-derived error, three experimental results show that a common pattern came from CS, where the systematic error originates from different local protein densities, which modulates the binding probability between DNA chains. Third, we suggested an iterative method to evaluate the contribution of DC, where DC is affected severely by the experimental setup, but we were unable to find a common pattern across the three experiments.

Many proteins participate in duplicating and packing DNA chains and thus yield different chromatin states. We provided regression coefficients for CS, which is related to the density of proteins, that link nearby DNA chains, over various chromatin states. Negative coefficients for active, inactive promoter regions indicate a lower density of DNA-binding proteins. This lower protein density supports the nucleosome depletion in promoter regions [16, 24], but the results are also controversial regarding the coefficient of the weak promoter, which has a small positive value that deviates from the expected pattern. Contrary to the obvious artifacts such as the reference genome-derived error and restriction enzyme-derived errors, the abundance caused by CS is a property of the nucleus. However, CS hinders the correct interpretation of contact frequencies into spatial distances; thus, we also removed the abundance caused by CS in the normalization process of contact frequency maps.

In previous studies, it has been assumed that better correlation between normalized contact frequency maps proves a better normalization method; however, we showed in this study that a better correlation does not always ensure a better normalization method. Here, we suggested a new normalization method. Although, the new method did not yield a better performance than that of the previous method, the newly-suggested correction factors showed better correlation with the coverage values, meaning that the new method is more efficient in removing the systematic errors.

## Supporting Information

S1 Fig A new mappability score. (A) Average coverage values classified by the old MS were plotted as open circles, open triangles, and open squares for three different experiments. The linear fitting results for each data are plotted as solid lines. (B) The genomic distances of mapping positions of paired-end reads were collected. The distribution of the distances depends on experimental methods. (C) The average coverage values classified by the new MS were plotted. (D) The standard deviations of the coverage values classified by both MSs were plotted, where open figures denote the result using the old MS, and solid figures denote the result using the new MS.
(TIF)

S2 Fig VIF for explanatory variables. For each experiment, we randomly divided all fragment ends into 10 groups. VIFs of the 616 explanatory variables were evaluated for each group using R. The mean values of VIFs were plotted as solid circles with error bars.
(TIF)

S3 Fig Experimental setup affects the type of contacts. Five categories of contacts, *cis*/*trans*-contact, self-circle, single-end, and dangling end can be easily classified in the analysis of paired-end reads. (A-E) Average frequencies for each category were evaluated according to their FL. (F) Average coverage values according to FL are shown. The right panel of each category shows normalized values by the maximum value.
(TIF)

S4 Fig Two entangled systematic errors. Multiple linear regressions were performed to evaluate the correlation coefficient between explanatory variables and coverage values. (A) The results for DC are plotted as solid figures. For comparison, results for the explanatory variables prepared by the AFE 500-bp sequences are plotted as open figures. (B) The results for DR are plotted as solid figures. Here, circles, triangles, and squares denote HH, HN, and TH data, respectively.
(TIF)

S5 Fig Regression coefficients of CS for individual experimental results. The regression coefficients for CS were measured individually for three sets of experimental results: HH, HN, and TH data. For each experimental result, standard deviations of coefficients were measured using 10 sets of explanatory variables generated by systematically extending BFE sequences.
(TIF)

S6 Fig Contact frequency maps between chromosomes 1 and 2. Contact frequency maps between chromosomes 1 and 2 were prepared based on 5-Mbp segments and normalized using two normalization methods. The intensity of the red color represents a natural logarithm of contact frequency.
(TIF)

S7 Fig Contact frequency maps. Contact frequency maps for HH, HN, and TH data were prepared based on 1-Mbp segments and normalized using two normalization methods. The intensity of the red color represents a natural logarithm of contact frequency.
(TIF)

S8 Fig Distribution of contact frequencies in contact frequency maps. For three different data sizes of HH data, contact frequency maps with 1-Mb segment sizes were prepared. Histograms of contact frequencies for each map were evaluated and normalized to yield probability distributions. For a data size of 5,000,000 points, the experimental probability distributions are fitted (a) using seven linearly combined Poisson distribution functions for *cis*-contacts and (b) using three linearly combined Poisson distribution functions for *trans*-contacts. The fitting curves were used to predict the probability distributions of different data sizes (c) for *cis*-contacts and (d) for *trans*-contacts.
(TIF)

S1 Text Detailed equations for ULC evaluation. (DOC)
