# Supplementary figures and images for: Identifying and Reducing Systematic Errors in Chromosome Conformation Capture Data
Source: PLoS One. 2015 Dec 30;10(12):e0146007. doi: 10.1371/journal.pone.0146007 (PMC4696798; doi:10.1371/journal.pone.0146007)

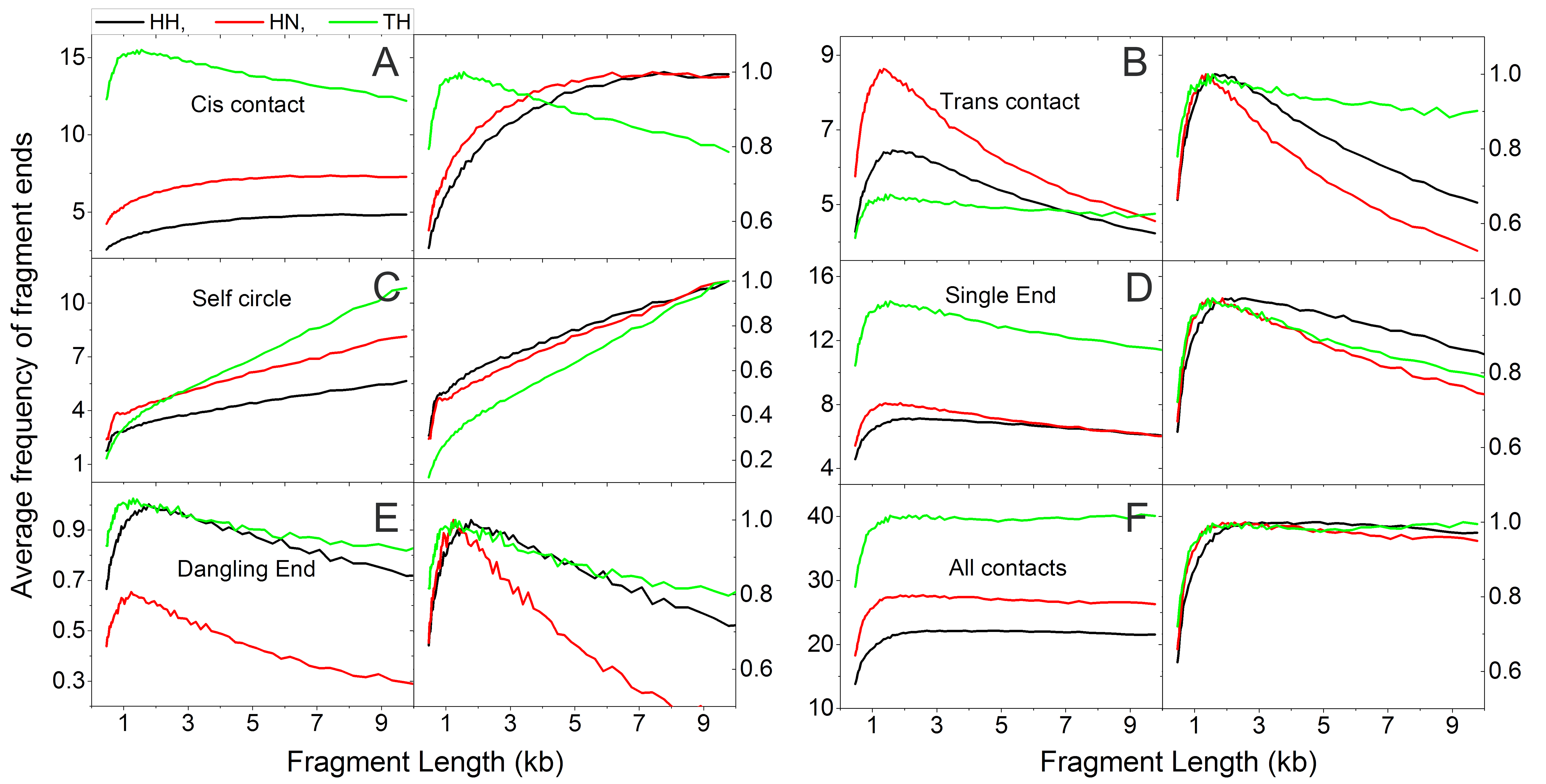

Supplement: S3 Fig — Five categories of contacts, cis/trans-contact, self-circle, single-end, and dangling end can be easily classified in the analysis of paired-end reads. (A-E) Average frequencies for each category were evaluated according to their FL. (F) Average coverage values according to FL are shown. The right panel of each category shows normalized values by the maximum value. (TIF) [file pone.0146007.s003.tif]

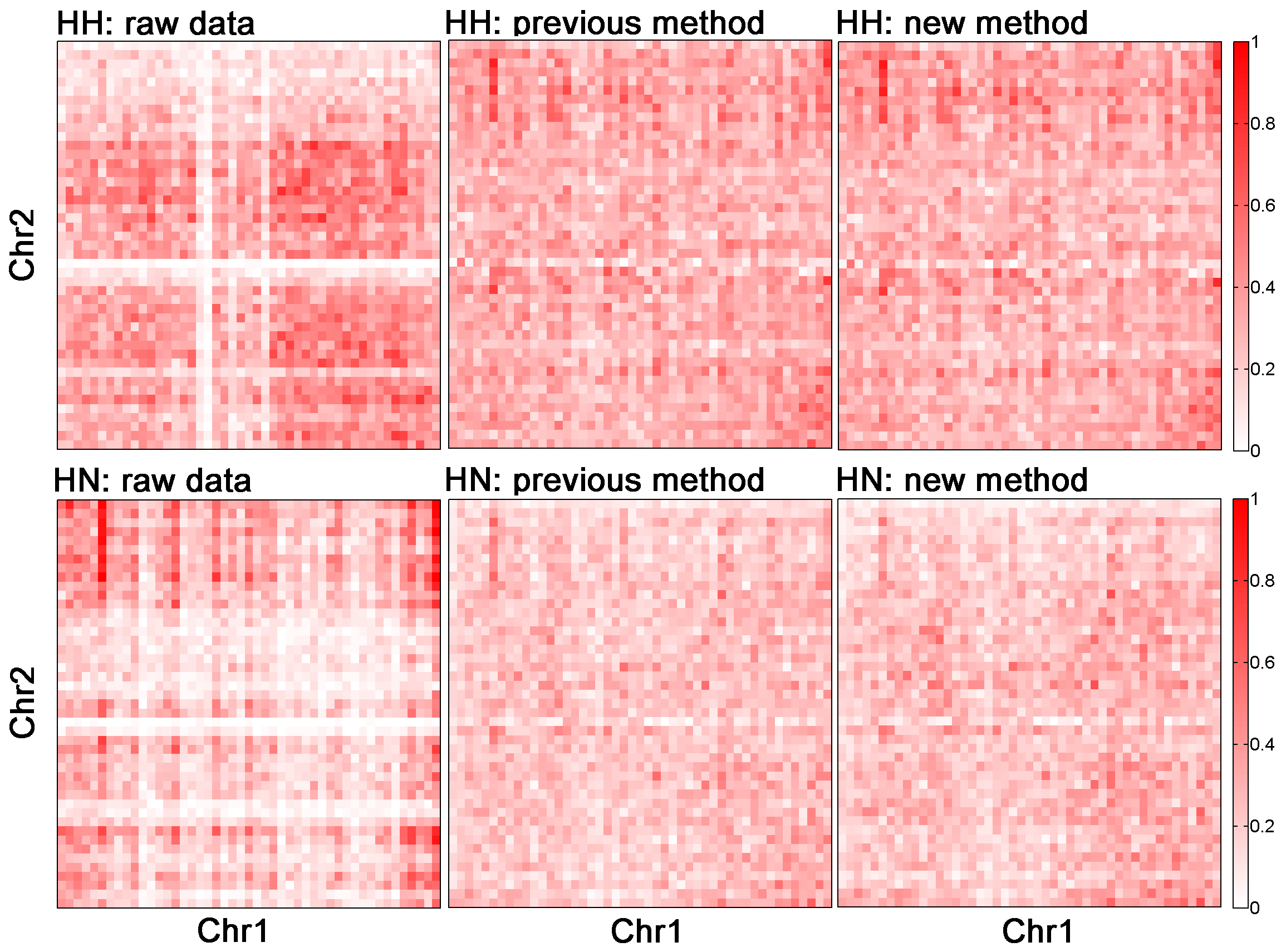

Supplement: S6 Fig — Contact frequency maps between chromosomes 1 and 2 were prepared based on 5-Mbp segments and normalized using two normalization methods. The intensity of the red color represents a natural logarithm of contact frequency. (TIF) [file pone.0146007.s006.tif]

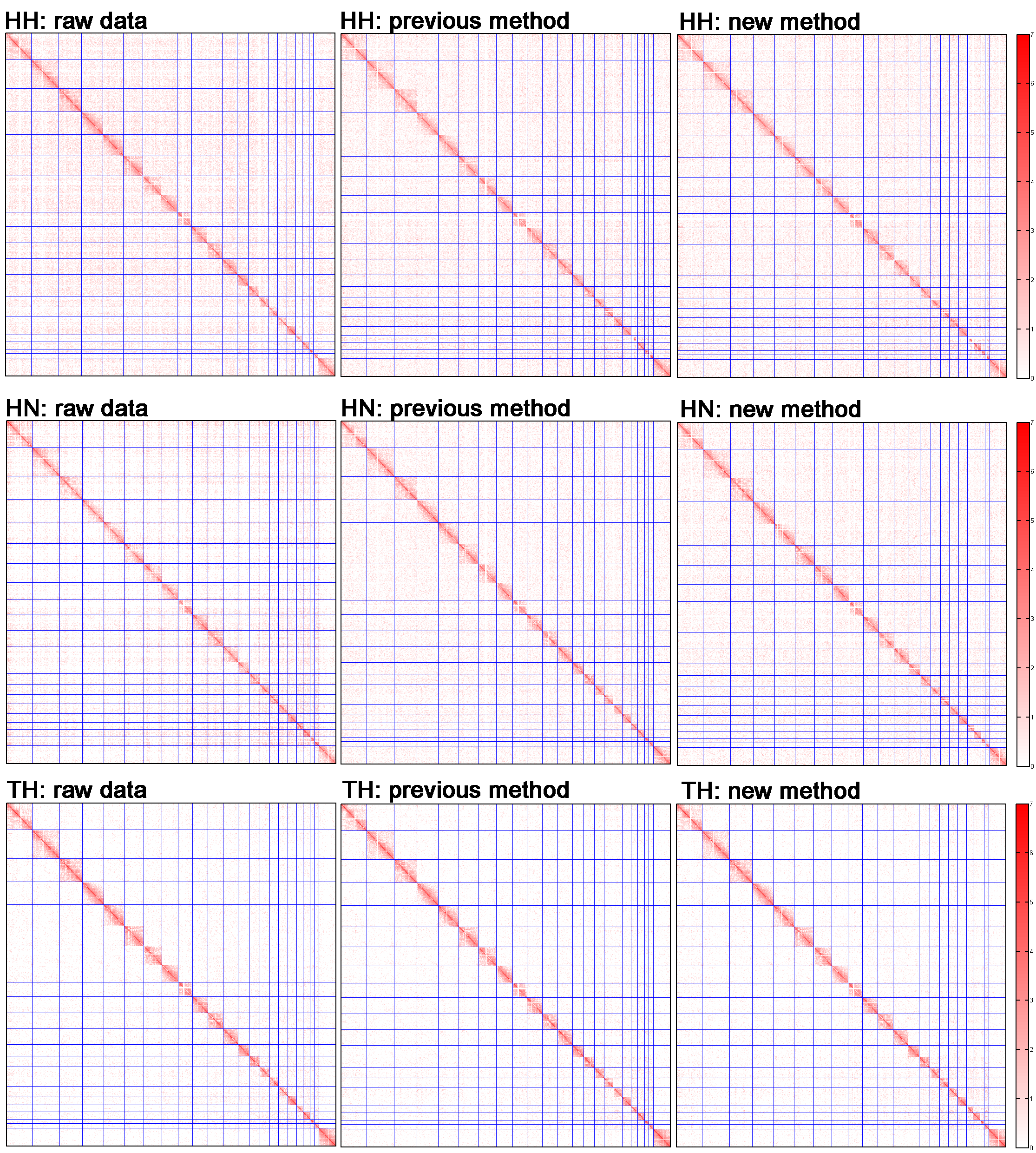

Supplement: S7 Fig — Contact frequency maps for HH, HN, and TH data were prepared based on 1-Mbp segments and normalized using two normalization methods. The intensity of the red color represents a natural logarithm of contact frequency. (TIF) [file pone.0146007.s007.tif]
