# Supplementary material for: Identifying and Reducing Systematic Errors in Chromosome Conformation Capture Data
Source: PLoS One. 2015 Dec 30;10(12):e0146007. doi: 10.1371/journal.pone.0146007 (PMC4696798; doi:10.1371/journal.pone.0146007)
Supplement: S1 Text — (DOC) [file pone.0146007.s009.doc]

**Supporting Information**

**Upper limit of correlation(ULC) of contact frequency maps**

A contact frequency between specific two genomic loci in a contact frequency map is determined by counting the number of paired sequence reads connecting the two loci. If we know the exact probabilities of all individual contacts, it is possible to calculate Pearson's correlation coefficient between contact frequency maps when repeating the same experiment. Let us assume that a $\left\{i,j\right\}$th contact has *m_ij_* contact frequency and *p_ij_* probability. The probability for selecting *m_ij_* contacts among the *N* total paired reads can be given as a probability expressed as following binomial distribution function:

$B\left(m_{ij};N,p_{ij}\right)=\left(\begin{matrix} N \\ m_{ij} \end{matrix}\right)\cdot p_{ij}^{m_{ij}}\cdot\left(1-p_{ij}\right)^{N-m_{ij}}$.

Contrary to the invariable probability, the contact frequency can be changed when repeating the same experiments. Mean and variance of the $\left\{i,j\right\}$th contact frequencies can be calculated by the following equations:

$$\left\langle m_{ij}\right\rangle=\sum_{m_{ij}=0}^{N} m_{ij}\cdot B\left(m_{ij};N,p_{ij}\right)=N\cdot p_{ij}$$

$\left\langle m_{ij}^{2}\right\rangle=\sum_{m_{ij}=0}^{N} m_{ij}^{2}\cdot B\left(m_{ij};N,p_{ij}\right)=N\cdot p_{ij}\cdot\left(N\cdot p_{ij}+1-p_{ij}\right)$,

where denotes the mean value.

To evaluate Pearson's correlation coefficient of off-diagonal elements between two contact frequency maps, we need another contact frequency map. Let us consider that the other map has *n_ij_* contact frequency at the same contact. Average properties of the number of points of the other map will be the same as the previous contact frequency as $\left\langle n_{ij}\right\rangle=\left\langle m_{ij}\right\rangle$ and $\left\langle n_{ij}^{2}\right\rangle=\left\langle m_{ij}^{2}\right\rangle$. Mean value for the correlation between the two contact frequencies is

$$\left\langle m_{ij}\cdot n_{ij}\right\rangle=\sum_{m_{ij}=0}^{N} \sum_{n_{ij}=0}^{N} m_{ij}\cdot n_{ij}\cdot B\left(m_{ij};N,p_{ij}\right)\cdot B\left(n_{ij};N,p_{ij}\right)$$

$$=\left(N\cdot p_{ij}\right)^{2}$$

Based on the mean values for individual contacts, we evaluate Pearson's correlation coefficient between two contact frequency maps. Mean number of contact frequencies for all contacts is:

$$\left\langle m\right\rangle=\frac{1}{S}\sum_{\left\{i,j\right\}\in all contacts} \left\langle m_{ij}\right\rangle=\frac{1}{S}\sum_{\left\{i,j\right\}\in all contacts} N\cdot p_{ij}$$

where *S* denotes total number of contacts in a contact frequency map. The variance and correlation can be easily evaluated as:

$$\left\langle m^{2}\right\rangle=\frac{1}{S}\sum_{\left\{i,j\right\}\in all contacts} \left\langle m_{ij}^{2}\right\rangle=\frac{1}{S}\sum_{\left\{i,j\right\}\in all contacts} N\cdot p_{ij}\cdot\left(N\cdot p_{ij}+1-p_{ij}\right)$$

$\left\langle m\cdot n\right\rangle=\frac{1}{S}\sum_{\left\{i,j\right\}\in all contacts} \left\langle m_{ij}\cdot n_{ij}\right\rangle=\frac{1}{S}\sum_{\left\{i,j\right\}\in all contacts} \left(N\cdot p_{ij}\right)^{2}$.

Here, $\left\langle n\right\rangle$ and $\left\langle n^{2}\right\rangle$ are equal to $\left\langle m\right\rangle$ and $\left\langle m^{2}\right\rangle$, respectively. Thus, Pearson's correlation coefficient for two contact frequency maps can be directly calculated from the above equations using following equation.

$$r=\frac{\left\langle m\cdot n\right\rangle-\left\langle m\right\rangle\cdot\left\langle n\right\rangle}{\sqrt{\left\langle m^{2}\right\rangle-\left\langle m\right\rangle^{2}}\sqrt{\left\langle n^{2}\right\rangle-\left\langle n\right\rangle^{2}}}$$

Because it is difficult to obtain the exact probability of each contact, we developed a method to reduce errors from the uncertainties in obtaining individual probabilities. The method starts with an assumption that we know the sum of counts (*o_ij_*) of the same two contacts *m_ij_* and *n_ij_*, or $o_{ij}=m_{ij}+n_{ij}$. A correlation coefficient between the two maps can be calculated by following methods:

$$\left\langle m_{ij}\right\rangle=\left\langle n_{ij}\right\rangle=\sum_{m_{ij}=0}^{o_{ij}} m_{ij}\cdot B\left(m_{ij};o_{ij},1/2\right)=o_{ij}/2$$

$$\left\langle m_{ij}^{2}\right\rangle=\left\langle n_{ij}^{2}\right\rangle=\sum_{m_{ij}=0}^{o_{ij}} m_{ij}^{2}\cdot B\left(m_{ij};o_{ij},1/2\right)=\frac{1}{4}o_{ij}\cdot\left(o_{ij}+1\right)$$

$$\left\langle m_{ij}\cdot n_{ij}\right\rangle=\sum_{m_{ij}=0}^{o_{ij}} m_{ij}\cdot\left(o_{ij}-m_{ij}\right)\cdot B\left(m_{ij};o_{ij},1/2\right)$$

$=\frac{1}{4}o_{ij}\cdot\left(o_{ij}-1\right)$.

Mean and variance values of all contacts between the two maps is given as:

$$\left\langle m\right\rangle=\left\langle n\right\rangle=\frac{1}{S}\sum_{\left\{i,j\right\}\in all contacts} \left\langle m_{ij}\right\rangle=\frac{1}{S}\sum_{\left\{i,j\right\}\in all contacts} o_{ij}/2=\left\langle o\right\rangle/2$$

$\left\langle m^{2}\right\rangle=\left\langle n^{2}\right\rangle=\frac{1}{S}\sum_{\left\{i,j\right\}\in all contacts} \left\langle m_{ij}^{2}\right\rangle=\frac{1}{4S}\sum_{\left\{i,j\right\}\in all contacts} o_{ij}\cdot\left(o_{ij}+1\right)=\frac{\left\langle o^{2}\right\rangle+\left\langle o\right\rangle}{4}$.

From the above equations, the correlation of contact frequencies between the maps is given as:

$\left\langle m\cdot n\right\rangle=\frac{1}{S}\sum_{\left\{i,j\right\}\in all contacts} \left\langle m_{ij}\cdot n_{ij}\right\rangle=\frac{\left\langle o^{2}\right\rangle-\left\langle o\right\rangle}{4}=\left\langle m^{2}\right\rangle-\left\langle m\right\rangle$.

Using Eq. , Pearson's correlation coefficient is given as

$$r=\frac{\left\langle m\cdot n\right\rangle-\left\langle m\right\rangle^{2}}{\left\langle m^{2}\right\rangle-\left\langle m\right\rangle^{2}}=\frac{\left\langle m^{2}\right\rangle-\left\langle m\right\rangle^{2}-\left\langle m\right\rangle}{\left\langle m^{2}\right\rangle-\left\langle m\right\rangle^{2}}=1-\frac{\left\langle m\right\rangle}{\left\langle m^{2}\right\rangle-\left\langle m\right\rangle^{2}}=1-\frac{\left\langle m\right\rangle}{Var\left(m\right)}$$

where *r* denotes Pearson's correlation coefficient. $\left\langle m\right\rangle$ and $Var\left(m\right)$ denote a mean and variance of all contact frequencies, respectively. This equation converts the problem for finding individual contact probabilities into an equation for calculating the mean and variance of all contact frequencies.

**Prediction of UCL for a larger dataset**

There are many contacts within contact frequency maps. Therefore, we make another assumption that there are many contacts with the similar real probability in a contact frequency map. For a group of contacts with the same probability, the contact frequencies will show a Poisson distribution. To verify the robustness of the assumption, we fit the histogram of contact frequencies using multiple Poisson distribution functions. In S8 Fig, populations are plotted as solid circles according to their contact frequencies for *cis*-contact and for *trans*-contact, respectively. For the data size of 5,000,000, the populations are fitted using seven Poisson distribution functions for *cis*-contacts and three Poisson distribution functions for *trans*-contacts. Using the fitted Poisson functions, we can exactly predict the population distribution of contact frequencies for a smaller size and larger size contact frequency maps. Based on this observation, we predict the Pearson's correlation coefficient for the contact frequency map with an arbitrary data size. Let us assume that $f\left(m;N\right)$ is the normalized histogram of the contact frequencies for data size of *N*. Pearson's correlation coefficient for $\alpha\cdot N$ data size can be evaluated as:

$$f\left(m;\alpha\cdot N\right)=\sum_{i} a_{i}\cdot P\left(m;\alpha\cdot\lambda_{i}\right)$$

$$\left\langle m\right\rangle=\left\langle n\right\rangle=\sum_{m=0}^{\infty} m\cdot f\left(m;\alpha\cdot N\right)$$

$$=\sum_{i} \sum_{m=0}^{\infty} a_{i}\cdot m\cdot P\left(m;\alpha\cdot\lambda_{i}\right)=\alpha\cdot\sum_{i} a_{i}\cdot\lambda_{i}=\alpha\cdot\left\langle m\right\rangle_{N}$$

$$\left\langle m^{2}\right\rangle=\left\langle n^{2}\right\rangle=\sum_{m=0}^{\infty} m^{2}\cdot f\left(m;\alpha\cdot N\right)$$

$$=\sum_{i} \sum_{m=0}^{\infty} a_{i}\cdot m^{2}\cdot P\left(m;\alpha\cdot\lambda_{i}\right)=\sum_{i} a_{i}\cdot\alpha\cdot\lambda_{i}\cdot\left(\alpha\cdot\lambda_{i}+1\right)$$

$=\alpha^{2}\cdot\left\langle m^{2}\right\rangle_{N}-\alpha\cdot\left(\alpha-1\right)\cdot\left\langle m\right\rangle_{N}$,

where *λ_i_* and *a_i_* denote the mean value and portion of the *i*th Poisson distribution function, respectively. denotes a mean value for data size of *N*. From Eq. , Pearson's correlation coefficient for the larger data size as:

$$r=1-\frac{\left\langle m\right\rangle_{N}}{\alpha\cdot\left(\left\langle m^{2}\right\rangle_{N}-\left\langle m\right\rangle_{N}^{2}\right)+\left(1-\alpha\right)\left\langle m\right\rangle_{N}}=1-\frac{\left\langle m\right\rangle_{N}}{\alpha\cdot Var\left(m\right)_{N}+\left(1-\alpha\right)\left\langle m\right\rangle_{N}}$$

**UCLs for normalized contact frequency maps**

Because of various systematic errors, normalization of a contact frequency map is necessary. If we assume a normalization factor of $\left\{i,j\right\}$th contact as $\gamma_{ij}$ then correlation coefficient can be evaluated as:

$$\left\langle\hat{m}_{ij}\right\rangle=\left\langle\hat{n}_{ij}\right\rangle=\sum_{m_{ij}=0}^{o_{ij}} \frac{m_{ij}}{\gamma_{ij}}\cdot B\left(m_{ij};o_{ij},1/2\right)=\frac{o_{ij}}{2\gamma_{ij}}$$

$$\left\langle\hat{m}_{ij}^{2}\right\rangle=\left\langle\hat{n}_{ij}^{2}\right\rangle=\sum_{m_{ij}=0}^{o_{ij}} \frac{m_{ij}^{2}}{\gamma_{ij}^{2}}\cdot B\left(m_{ij};o_{ij},1/2\right)=\frac{1}{4\gamma_{ij}^{2}}o_{ij}\cdot\left(o_{ij}+1\right)$$

$$\left\langle\hat{m}_{ij}\cdot\hat{n}_{ij}\right\rangle=\sum_{m_{ij}=0}^{o_{ij}} \frac{m_{ij}\cdot\left(o_{ij}-m_{ij}\right)}{\gamma_{ij}^{2}}\cdot B\left(m_{ij};o_{ij},1/2\right)$$

$$=\frac{1}{4\gamma_{ij}^{2}}o_{ij}\cdot\left(o_{ij}-1\right)$$

where $\hat{m}_{ij}$ denotes normalized contact frequency. Mean and variance of all contacts between the two maps are given as:

$$\left\langle\hat{m}\right\rangle=\left\langle\hat{n}\right\rangle=\frac{1}{S}\sum_{\left\{i,j\right\}\in all contacts} \left\langle\hat{m}_{ij}\right\rangle=\frac{1}{S}\sum_{\left\{i,j\right\}\in all contacts} \frac{o_{ij}}{2\gamma_{ij}}=\frac{\left\langle\hat{o}\right\rangle}{2}$$

$\left\langle\hat{m}^{2}\right\rangle=\left\langle\hat{n}^{2}\right\rangle=\frac{1}{S}\sum_{\left\{i,j\right\}\in all contacts} \left\langle\hat{m}_{ij}^{2}\right\rangle=\frac{1}{4S}\sum_{\left\{i,j\right\}\in all contacts} \frac{o_{ij}\cdot\left(o_{ij}+1\right)}{\gamma_{ij}^{2}}=\frac{\left\langle\hat{o}^{2}\right\rangle+\left\langle\hat{o}/\gamma\right\rangle}{4}$.

From the above equations, the correlation of contact frequencies between the maps is given as:

$$\left\langle\hat{m}\cdot\hat{n}\right\rangle=\frac{1}{S}\sum_{\left\{i,j\right\}\in all contacts} \left\langle\hat{m}_{ij}\cdot\hat{n}_{ij}\right\rangle=\frac{1}{4S}\sum_{\left\{i,j\right\}\in all contacts} \frac{o_{ij}\cdot\left(o_{ij}-1\right)}{\gamma_{ij}^{2}}$$

$$=\frac{\left\langle\hat{o}^{2}\right\rangle-\left\langle\hat{o}/\gamma\right\rangle}{4}=\left\langle\hat{m}^{2}\right\rangle-\left\langle\hat{m}/\gamma\right\rangle$$

with $\left\langle\hat{m}/\gamma\right\rangle=\frac{1}{S}\sum_{\left\{i,j\right\}\in all contacts} \left\langle\hat{m}_{ij}/\gamma_{ij}\right\rangle$.

Using Eq. , Pearson's correlation coefficient is given as:

$$r=\frac{\left\langle\hat{m}\cdot\hat{n}\right\rangle-\left\langle\hat{m}\right\rangle^{2}}{\left\langle\hat{m}^{2}\right\rangle-\left\langle\hat{m}\right\rangle^{2}}=\frac{\left\langle\hat{m}^{2}\right\rangle-\left\langle\hat{m}\right\rangle^{2}-\left\langle\hat{m}/\gamma\right\rangle}{\left\langle\hat{m}^{2}\right\rangle-\left\langle\hat{m}\right\rangle^{2}}=1-\frac{\left\langle\hat{m}/\gamma\right\rangle}{\left\langle\hat{m}^{2}\right\rangle-\left\langle\hat{m}\right\rangle^{2}}=1-\frac{\left\langle\hat{m}/\gamma\right\rangle}{Var\left(\hat{m}\right)}$$

**SI Reference**

1. Lieberman-Aiden E, van Berkum NL, Williams L, Imakaev M, Ragoczy T, et al. (2009) Comprehensive mapping of long-range interactions reveals folding principles of the human genome. *Science* 326: 289-293.

2. Kalhor R, Tjong H, Jayathilaka N, Alber F, Chen L (2012) Genome architectures revealed by tethered chromosome conformation capture and population-based modeling. *Nat Biotechnol* 30: 90-98.

3. Langmead B, Salzberg SL (2012) Fast gapped-read alignment with Bowtie 2. *Nat Methods* 9: 357-359.

4. Ernst J, Kheradpour P, Mikkelsen TS, Shoresh N, Ward LD, et al. (2011) Mapping and analysis of chromatin state dynamics in nine human cell types. *Nature* 473: 43-49
